# P-1581. Incidence of Cystitis and Vaginitis/Balanitis in US Veterans on Sodium/Glucose Cotransporter 2 Inhibitor Therapy for Diabetes

**DOI:** 10.1093/ofid/ofae631.1748

**Published:** 2025-01-29

**Authors:** Zeena Lobo, George Psevdos

**Affiliations:** Northport VA Medical Center, Northport, New York; Northport VA Medical Center, Northport, New York

## Abstract

**Background:**

Sodium glucose co-transporter 2 inhibitors (SGLT-2) are currently recommended for type 2 Diabetes, chronic kidney disease and atherosclerotic cardiovascular disease. Despite their known health benefits, a recent study within the Veterans Affairs (VA) Health care system found a relatively low rate of utilization for SGLT-2. Several studies have shown an association of SGLT-2 with increased risk for genital mycotic infections, cystitis and urosepsis. We evaluated the incidence of such infectious complications in US Veterans (VET)

**Table 1.**
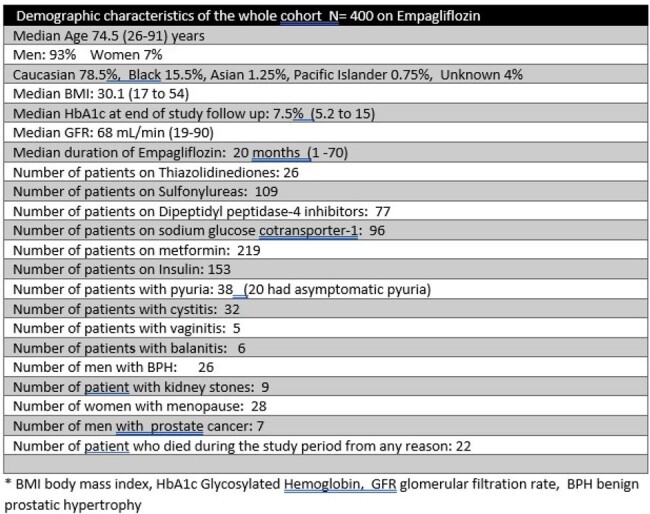
Demographic Characteristics of the Cohort

**Methods:**

Retrospective chart review of VET at Northport VA Medical Center who had received at least six months of SGLT-2 empagliflozin (EMP) for diabetes. VET who received EMP for heart failure only were excluded. The study years were 2018-2023. Demographic data, urinalysis, urine cultures, medical charts were reviewed for documentation of cystitis, clinical diagnosis of vaginitis/balanitis and need for hospitalization for urosepsis

**Table 2.**
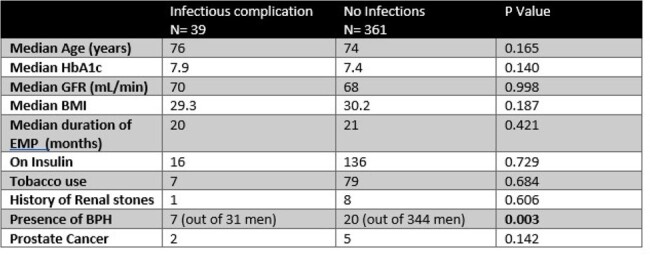
Comparison of the two groups: infectious vs. no infectious complications

**Results:**

400 VET were analyzed in the study period. The median age was 74.5 years. 78% were Caucasian, 15.5% black. 93% men. Table 1 shows the demographics of the cohort. There were 43 (10.75%) occasions of infectious complications: 32 (8%) cystitis (4 in women; one recurrent), 6 Balanitis, 5 fungal vaginitis (2 recurrent) . EMP was discontinued in all cases. 7/32 cystitis were clinical diagnoses without culture data. 6 cases of cystitis had more than 1 organism isolated. 2 VET were hospitalized due to urosepsis. There were no multidrug resistant organisms isolated (2 *E. coli* were resistant to ciprofloxacin) 1 case of balanitis in an HIV-infected man was cultured and grew *Trichoderma atroviride.* No cultures were obtained in cases of vaginitis. There were no differences in the two groups (infectious complications vs. noninfectious) in terms of age, HbA1c, BMI, GFR, duration of EMP treatment, use of insulin; However BPH was more common in the infectious group. See table 2. Table 3 lists the organisms isolated in urine cultures. No patient died from infectious complications

**Table 3.**
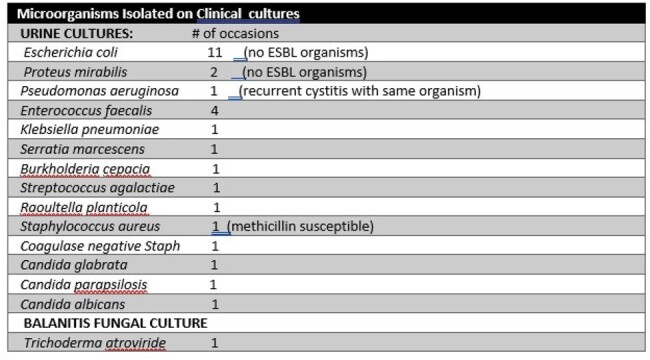
Organisms isolated in clinical specimens

**Conclusion:**

The incidence of infectious complications in our diabetic VET due to EMP was overall low, with cystitis being at 8%, and observed more in men with prostatomegaly. *E. coli* was the most frequent isolate in urine cultures but no multidrug resistant organisms seen

**Disclosures:**

**All Authors**: No reported disclosures

